# Extended Follow‐Up Analysis of First‐Line Atezolizumab in Extensive‐Stage Small Cell Lung Cancer: A Real‐World Multicenter Prospective Cohort Study

**DOI:** 10.1111/1759-7714.70201

**Published:** 2025-12-11

**Authors:** Yechan Song, Myeong Geun Choi, Yeon Joo Kim, Jae Cheol Lee, Wonjun Ji, In‐Jae Oh, Sung Yong Lee, Seong Hoon Yoon, Shin Yup Lee, Jeong Eun Lee, Eun Young Kim, Ho Young Kim, Chang‐Min Choi

**Affiliations:** ^1^ Department of Pulmonary and Critical Care Medicine Asan Medical Center, University of Ulsan College of Medicine Seoul Korea; ^2^ Division of Pulmonary and Critical Care Medicine, Department of Internal Medicine Ewha Womans University Mokdong Hospital, Ewha Womans University College of Medicine Seoul Korea; ^3^ Department of Pulmonology Nowon Eulji Medical Center, Eulji University School of Medicine Seoul Korea; ^4^ Department of Oncology Asan Medical Center, University of Ulsan College of Medicine Seoul Korea; ^5^ Department of Internal Medicine Chonnam National University Hwasun Hospital, Chonnam National University Medical School Hwasun Korea; ^6^ Division of Pulmonary, Allergy, and Critical Care Medicine, Department of Internal Medicine Korea University Guro Hospital, Korea University College of Medicine Seoul Korea; ^7^ Division of Pulmonology, Allergy, and Critical Care Medicine, Department of Internal Medicine Pusan National University Yangsan Hospital Yangsan Korea; ^8^ Department of Internal Medicine Kyungpook National University, School of Medicine Daegu Korea; ^9^ Division of Pulmonology, Department of Internal Medicine Chungnam National University College of Medicine Daejeon Korea; ^10^ Division of Pulmonary and Critical Care Medicine, Department of Internal Medicine Severance Hospital, Yonsei University College of Medicine Seoul Korea; ^11^ Division of Hematology and Oncology, Department of Internal Medicine Hallym University Sacred Heart Hospital Anyang Korea

**Keywords:** atezolizumab, real‐world, small cell lung carcinoma

## Abstract

**Background:**

We previously reported the short‐term real‐world effectiveness and safety of first‐line atezolizumab combined with chemotherapy in patients with extensive‐stage small cell lung cancer (ES‐SCLC). This study provides an updated analysis of the effectiveness, prognostic factors, and subsequent treatment patterns in first‐line immunochemotherapy.

**Methods:**

This prospective multicenter observational study enrolled patients with ES‐SCLC, diagnosed at seven university hospitals throughout Korea, between June 2021 and August 2022. Primary outcomes were 1‐year overall survival (OS) and progression‐free survival (PFS), whereas secondary outcomes included OS, objective response rate, disease control rate, second progression‐free survival, and safety, evaluated based on established clinical guidelines.

**Results:**

A total of 100 ES‐SCLC patients (median age, 69 years) were enrolled, with a median follow‐up duration of 26.0 months. The median PFS and OS were 6.2 and 17.1 months, respectively, with a 1‐year OS rate of 62.5%. Favorable prognostic factors for OS included partial response (PR) and stable disease (SD) as the best responses (SD: hazard ratio (HR), 0.79; PR: HR, 0.38) and a longer platinum‐free interval (HR 0.84). Brain radiotherapy significantly improved OS in patients with brain metastases, whereas thoracic radiotherapy during first‐line treatment tended to prolong survival in patients who responded to systemic treatment. Patients receiving second‐line treatment after progression presented a significantly longer OS than did those receiving only best supportive care.

**Conclusion:**

This study outlined the real‐world effectiveness and safety of first‐line atezolizumab immunochemotherapy for ES‐SCLC patients over an extended follow‐up, noting that local treatment and post‐progression therapy were associated with improved survival.

## Graphical abstract

This multicenter prospective cohort study provides real‐world evidence on the favorable effectiveness and safety of front‐line atezolizumab combined with chemotherapy for patients with extensive‐stage small cell lung cancer over an extended follow‐up period, highlighting the potential value of local treatment and post‐progression therapy for eligible patients.

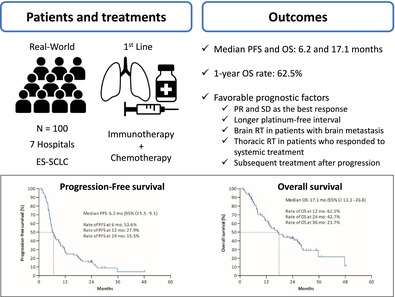

## 1 Introduction

Currently, anti‐programmed death‐ligand 1 (PD‐L1) blockade in combination with a platinum agent and etoposide is the standard first‐line management of extensive‐stage small cell lung cancer (ES‐SCLC). Multiple randomized phase III studies have shown that adding an anti‐PD‐L1 blockade to a standard platinum–etoposide backbone, while continuing immunotherapy as maintenance, improved both progression‐free survival (PFS) and overall survival (OS) when compared to chemotherapy alone [1, 2]. IMpower133, a global double‐blind, phase III, randomized, placebo‐controlled trial, compared the outcomes of ES‐SCLC patients treated with atezolizumab in addition to traditional platinum and etoposide chemotherapy with those of patients treated only with conventional chemotherapy. The IMpower133 study showed that the atezolizumab‐treated group demonstrated a significant improvement in both the median OS and PFS (median OS 12.3 vs. 10.3 months; hazard ratio (HR) 0.76; *p* = 0.0154) regardless of PD‐L1 immunohistochemistry or blood tumor mutation burden status [3, 4].

Although the atezolizumab and chemotherapy combination has been established as the standard first‐line regimen for ES‐SCLC based on these pivotal trials, the applicability of this combination in unselected real‐world populations remains insufficiently characterized. Several real‐world studies evaluating the effectiveness and safety of this regimen have since been published [5, 6, 7, 8, 9, 10, 11], with the results being generally consistent with those of IMpower133. However, most of these studies were retrospective in nature, and only a limited number of prospective studies have assessed long‐term outcomes of atezolizumab plus platinum–etoposide immunochemotherapy in this setting.

Our team previously conducted a multicenter prospective cohort study with a median follow‐up of 13.2 months that investigated the short‐term real‐world effectiveness and safety of atezolizumab combined with chemotherapy [11]. Building on that study, the current analysis provides updated findings with extended follow‐up data from the same cohort. Therefore, this multicenter prospective study aimed to evaluate the effectiveness and safety of first‐line chemoimmunotherapy in real‐world clinical practice and to identify prognostic factors and treatment patterns that may help optimize the management of patients with ES‐SCLC.

## 2 Methods

### 2.1 Patient Inclusion Criteria and Study Design

This prospective observational multicenter study evaluated the real‐world clinical outcomes of atezolizumab combined with chemotherapy in ES‐SCLC patients. Prospective enrollment included patients initially diagnosed with ES‐SCLC at seven university hospitals across South Korea from June 2021 to August 2022. Details regarding patient enrollment have been published previously [11].

### 2.2 Treatment and Assessment

Patients were treated according to the prior clinical trial regimen and were maintained on atezolizumab until disease progression [3]. Tumor response re‐evaluation was conducted according to the recommendation of the Response Evaluation Criteria in Solid Tumors (RECIST) version 1.1. Adverse events were assessed according to the Common Terminology Criteria for Adverse Events version 5.0. Patients were censored at the final follow‐up or data cutoff date (i.e., August 31, 2024).

### 2.3 Outcomes

The primary outcomes were 1‐year OS rate and investigator‐assessed PFS. Secondary outcomes included OS, objective response rate (ORR), disease control rate (DCR), time to second objective disease progression‐free survival (PFS2), and safety.

PFS was defined as the duration from the first day of chemotherapy initiation to disease progression or death from any cause, whereas OS was defined as the duration from the first day of chemotherapy initiation to death from any cause. PFS2 was defined as the duration from the first day of second‐line chemotherapy initiation to the second disease progression or death from any cause, based on the recommendation of the European Medicines Agency [12]. Intracranial progression‐free survival (IC‐PFS) was defined as the time from the start of first‐line systemic therapy to intracranial disease progression. Meanwhile, for patients who received brain radiotherapy, post‐local treatment IC‐PFS was calculated from the initiation of brain RT to either intracranial progression or death. ORR was determined based on the RECIST 1.1 guidelines. DCR was defined as the percentage of patients with complete response, partial response (PR), or stable disease (SD) as the best response. Platinum‐free interval (PFI) was defined as the duration from the last day of first‐line platinum agent administration to disease progression. Data for thoracic radiotherapy (RT) were collected based on location and method. Data for brain metastases (BM) were collected based on the date of diagnosis and treatment method.

### 2.4 Statistical Analysis

Clinical characteristics were summarized using descriptive statistics. The Kaplan–Meier method was used to estimate the probability of OS, PFS, PFS2, and follow‐up duration. Survival outcomes were compared using Fisher's exact test and the log‐rank test. Multivariable Cox regression and multivariable logistic regression models were used to identify factors predicting survival outcomes. The mean for each group was compared using Student's *t*‐test. A *p*‐value of < 0.05 indicated a priori statistical significance. All analyses were performed using SPSS Statistics for Windows (version 25.0; IBM SPSS, Armonk, NY).

## 3 Results

### 3.1 Patient and Treatment Characteristics

A total of 100 patients with ES‐SCLC from seven centers were enrolled. Baseline patient characteristics, as summarized in our previously published study, are shown in Table S1.

### 3.2 Assessment of PFS, OS, and Effectiveness

**FIGURE 1:**
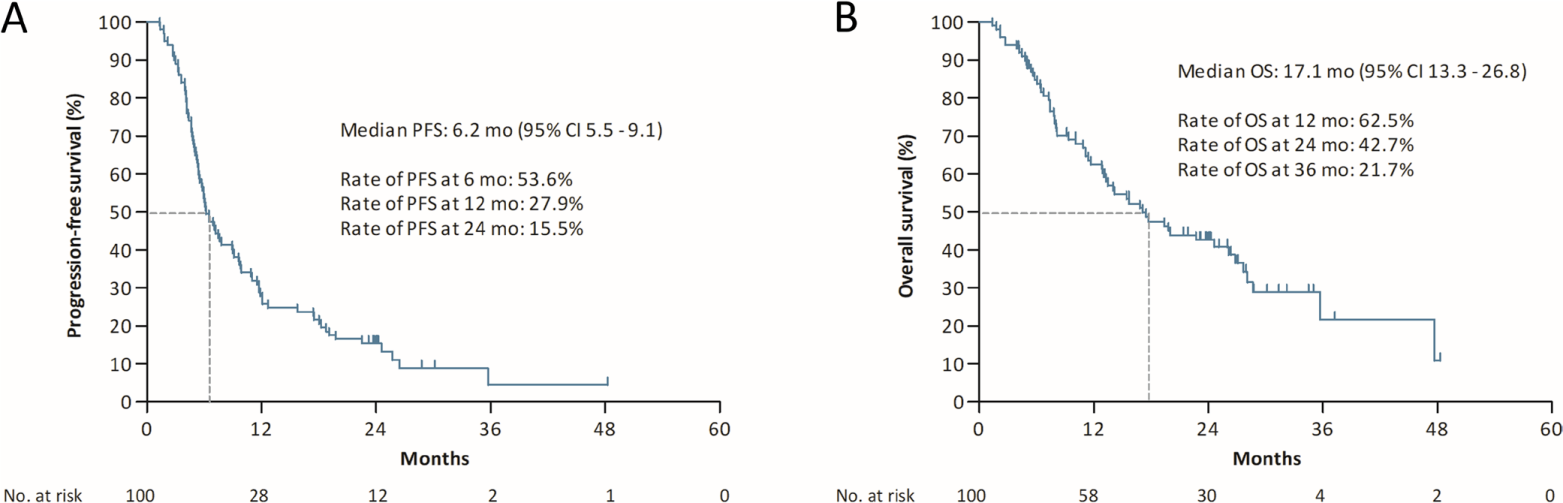
Kaplan–Meier curves for (A) progression‐free survival (PFS) and (B) overall survival (OS) in patients with small cell lung cancer receiving atezolizumab plus chemotherapy. 95% CI, 95% confidence interval.

The median follow‐up duration was 26.0 months (95% confidence interval (CI) 24.2–30.2) at the data cutoff date (Table 1). Among the included patients, 87.0% (*n* = 87) developed disease progression, whereas 61.0% (*n* = 61) were deceased by the data cutoff date. The median PFS was 6.2 months (95% CI 5.5–9.1) (Figure 1). PFS rates at 6, 12, and 24 months were 53.6%, 27.9%, and 15.5%, respectively. The median OS was 17.1 months (95% CI 13.3–26.8). Survival rates at 12, 24, and 36 months were 62.5%, 42.7%, and 21.7%, respectively. ORR was 75% (*n* = 75), whereas DCR was 91% (*n* = 91) (Table 1).

**TABLE 1:** Effectiveness of atezolizumab plus chemotherapy treatment.

Variable	Total patients (*n* = 100)	IMpower133 (*n* = 201)
Median follow‐up duration (months)	26.0 (24.2–30.2)	13.9 (N/E–N/E)
Best response		
Complete response	1 (1.0)	5 (2.5)
Partial response	74 (74.0)	116 (57.7)
Stable disease	16 (16.0)	42 (20.9)
Progressive disease	8 (8.0)	22 (10.9)
Not evaluated	1 (1.0)	16 (8.0)
Objective response rate (%)	75	60.2
Disease control rate (%)	91	78.6
Median PFS (months)	6.2 (5.5–9.1)	5.2 (4.4–5.6)
Median OS (months)	17.1 (13.3–26.8)	12.3 (10.8–15.9)
OS rate at 1 year (%)	62.5	51.7

*Note:* Values are presented as the median (95% confidence interval) or number (%) unless otherwise specified.

Abbreviations: 95% CI, 95% confidence interval; N/E, not evaluated; OS, overall survival; PFS, progression‐free survival.

### 3.3 Prognostic Factors for PFS and OS

The updated multivariable Cox analysis adjusted according to age, sex, and Eastern Cooperative Oncology Group Performance Status (ECOG PS) found that both SD and PR (best response) were more favorable prognostic factors for PFS (SD: adjusted HR, 0.20; *p* < 0.001; PR: adjusted HR, 0.20; *p* = 0.001) and OS (SD: adjusted HR, 0.79; *p* = 0.003; PR: adjusted HR, 0.38; *p* = 0.053) than progressive disease (Table 2). A longer PFI was identified as a favorable prognostic factor for OS (adjusted HR, 0.84, 95% CI 0.76–0.92; *p* < 0.001). Our updated survival analysis noted that thoracic RT and BM at diagnosis were not significantly associated with differences in survival outcomes.

**TABLE 2:** Multivariable Cox regression analysis for progression‐free survival and overall survival.

Variable	Progression‐free survival		Overall survival	
	aHR (95% CI)^a^	*p*	aHR (95% CI)^a^	*p*
Ever smoker	1.14 (0.50–2.61)	0.756	0.49 (0.18–1.39)	0.178
Thoracic RT	1.09 (0.68–1.75)	0.718	1.37 (0.78–2.40)	0.271
Thoracic RT during first‐line immunochemotherapy	0.92 (0.57–1.47)	0.718	0.73 (0.42–1.28)	0.271
BM (at diagnosis)	1.17 (0.71–1.94)	0.545	1.08 (0.60–1.94)	0.803
BM (entire treatment period)	0.94 (0.61–1.46)	0.788	1.12 (0.67–1.89)	0.664
Best response				
PD	Ref.	Ref.	Ref.	Ref.
SD	0.20 (0.09–0.46)	< 0.001	0.79 (0.13–0.66)	0.003
PR	0.20 (0.08–0.52)	0.001	0.38 (0.14–1.01)	0.053
PFI (days)	—	—	0.84 (0.76–0.92)	< 0.001
PFI > 6 months	—	—	0.18 (0.08–0.40)	< 0.001

Abbreviations: 95% CI, 95% confidence interval; aHR, adjusted hazard ratio; BM, brain metastasis; ECOG PS, Eastern Cooperative Oncology Group Performance Status; PD, progressive disease; PFI, platinum‐free interval; PR, partial response; Ref., reference; RT, radiotherapy; SD, stable disease.

^a^ Adjusted by age, sex, and ECOG PS.

Logistic analysis found that thoracic RT and a longer PFI were favorable factors for predicting long‐term responders (PFS > 6.2 months) and long‐term survivors (OS > 17.1 months), respectively (Table 3).

**TABLE 3:** Multivariable logistic regression analysis to predict long‐term responders and long‐term survivors.

Variable	Long‐term responders		Long‐term survivors	
	aOR (95% CI)^a^	*p*	aOR (95% CI)^a^	*p*
Ever smoker	1.11 (0.25–5.03)	0.890	0.32 (0.06–1.79)	0.190
Thoracic RT	3.52 (1.39–9.56)	0.010	1.79 (0.71–4.58)	0.217
Thoracic RT during first‐line immunochemotherapy	7.87 (2.49–31.20)	0.001	1.50 (0.53–4.22)	0.436
BM (at diagnosis)	1.35 (0.54–3.37)	0.520	1.00 (0.40–2.54)	0.997
BM (entire treatment period)	1.43 (0.63–3.21)	0.390	0.99 (0.43–2.26)	0.973
Best response				
PD	Ref.	Ref.	Ref.	Ref.
SD	N/E (N/E–N/E)	N/E	8.21 (0.61–109.87)	0.112
PR	N/E (N/E–N/E)	N/E	14.97 (0.92–244.94)	0.058
PFI (days)	1.18 (0.99–1.41)	0.060	1.01 (1.01–1.02)	0.001
PFI > 6 months	—	—	29.71 (5.48–161.10)	< 0.001

Abbreviations: 95% CI, 95% confidence interval; aOR, adjusted odds ratio; BM, brain metastasis; ECOG PS, Eastern Cooperative Oncology Group Performance Status; PD, progressive disease; PFI, platinum‐free interval; PR, partial response; Ref., reference; RT, radiotherapy; SD, stable disease.

^a^ Adjusted by age, sex, and ECOG PS.

### 3.4 Subgroup Analysis

**FIGURE 2:**
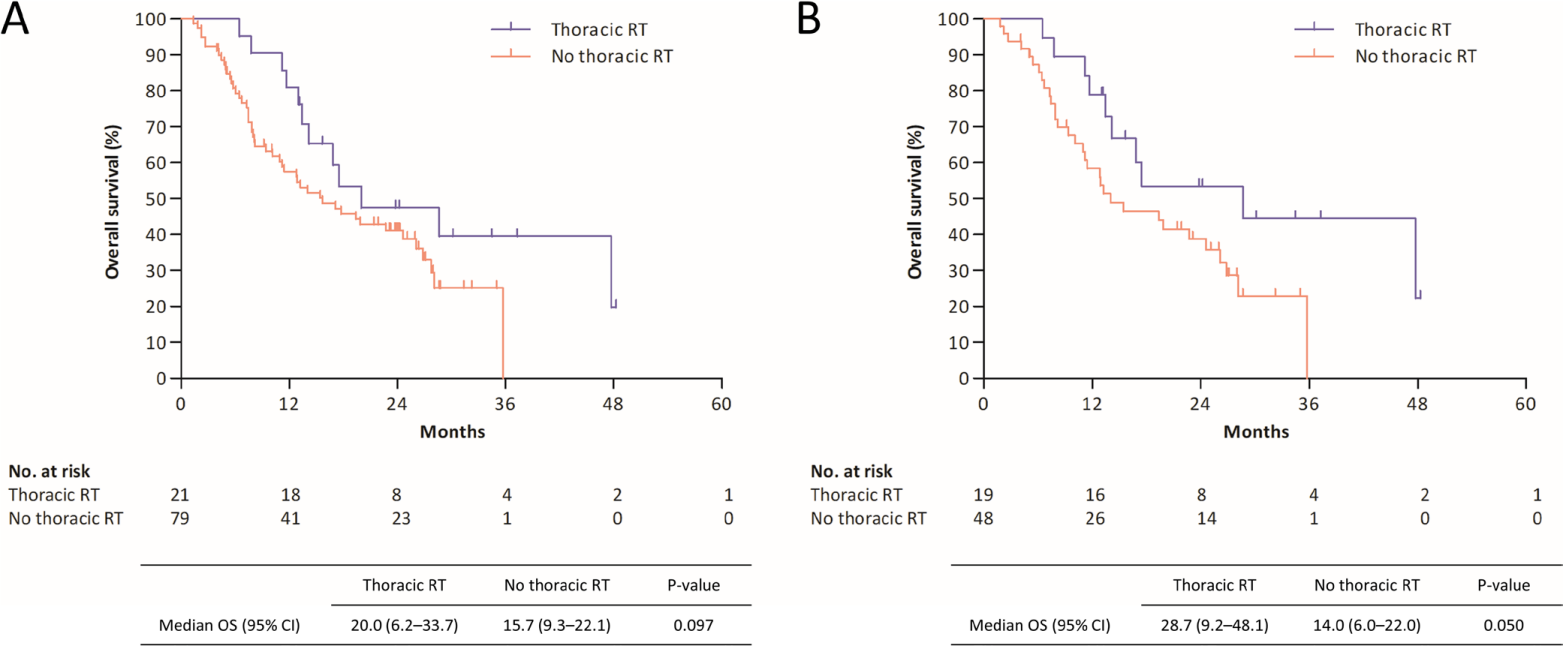
Survival analysis according to thoracic radiotherapy (RT) during first‐line immunochemotherapy among (A) all patients and (B) patients whose disease responded to systemic treatment. OS, overall survival; 95% CI, 95% confidence interval.

Throughout the study period, 30% of the patients (*n* = 30) received thoracic RT, with 70% (*n* = 21) of patients receiving thoracic RT during the first‐line treatment. For these 21 patients, the median interval from initiation of first‐line treatment to the start of thoracic RT was 3.6 months. No significant survival difference was observed between patients who did and did not receive thoracic RT during first‐line treatment; however, among patients who showed a response to first‐line atezolizumab, those who received concurrent thoracic RT tended to exhibit increased survival (median OS 28.7 vs. 14.0 months; *p* = 0.050; Figure 2).

**FIGURE 3:**
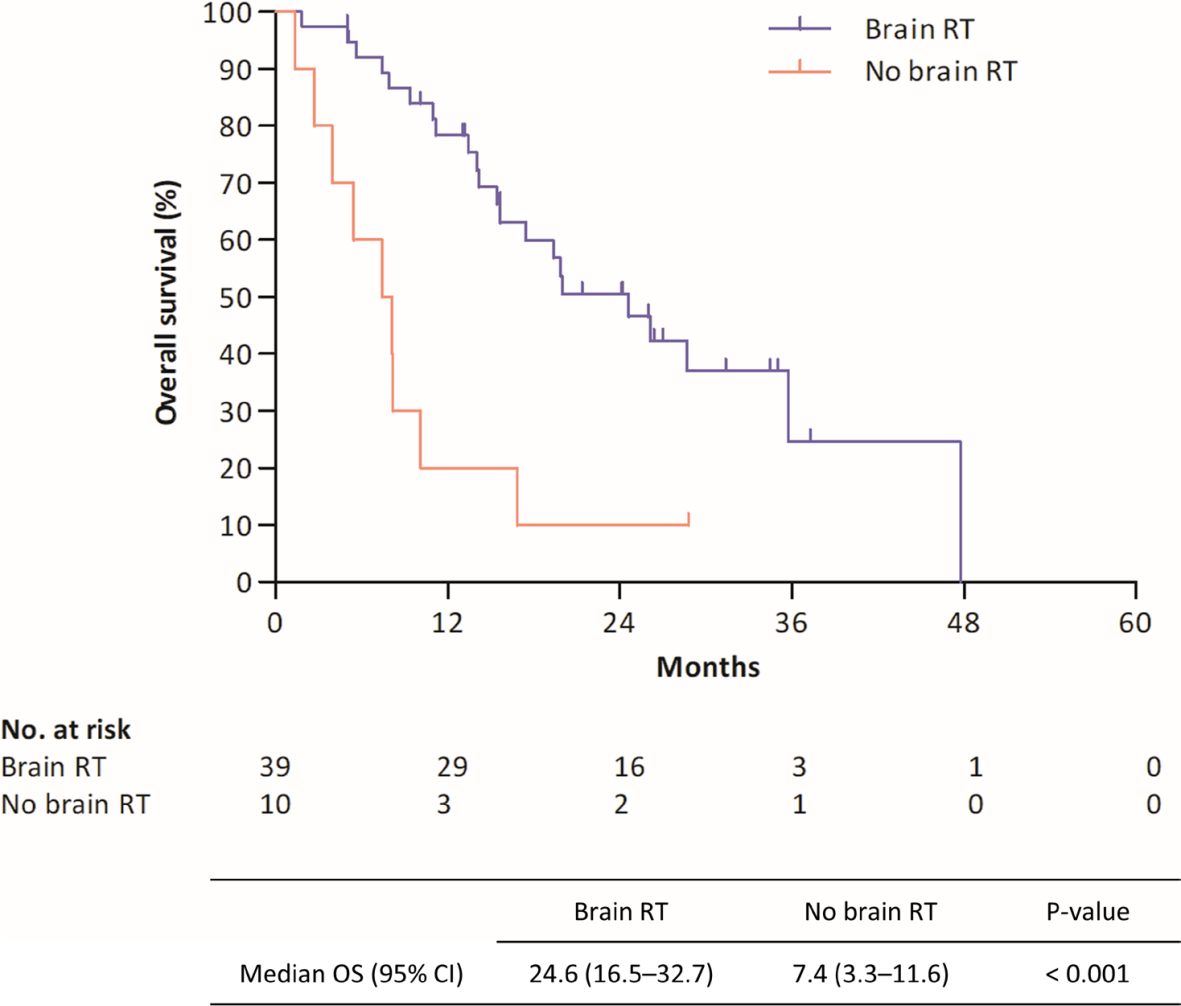
Survival analysis according to brain radiotherapy (RT) in patients with brain metastases. OS, overall survival; 95% CI, 95% confidence interval.

Among the analyzed patients, 26% (*n* = 26) had been initially diagnosed with BM, whereas 23% (*n* = 23) developed BM during the study period. Among those who were diagnosed with BM throughout the whole study period (*n* = 49), 79.6% (*n* = 39) received local brain treatment, all of whom received RT as the initial treatment (treatment method: 43.5% gamma knife radiosurgery; 5.2% Cyberknife, 46.2% whole‐brain RT (WBRT); 5.1% tomotherapy). For patients with baseline brain metastases, the intracranial progression‐free survival (IC‐PFS) was 9.3 months (95% CI 7.1–26.5). Patients who received brain radiotherapy—irrespective of the timing of brain metastasis—had a post‐local treatment IC‐PFS of 10.5 months (95% CI 7.1–13.9). Among the patients who had BM at diagnosis and those who developed BM during treatment, those who received brain RT experienced a longer OS than those who did not (median OS 24.6 vs. 7.4 months; *p* < 0.001; Figure 3).

The Kaplan–Meier curves for the outcomes of each variable are shown in Figures S1 and S2.

### 3.5 Subsequent Treatment Pattern

**FIGURE 4:**
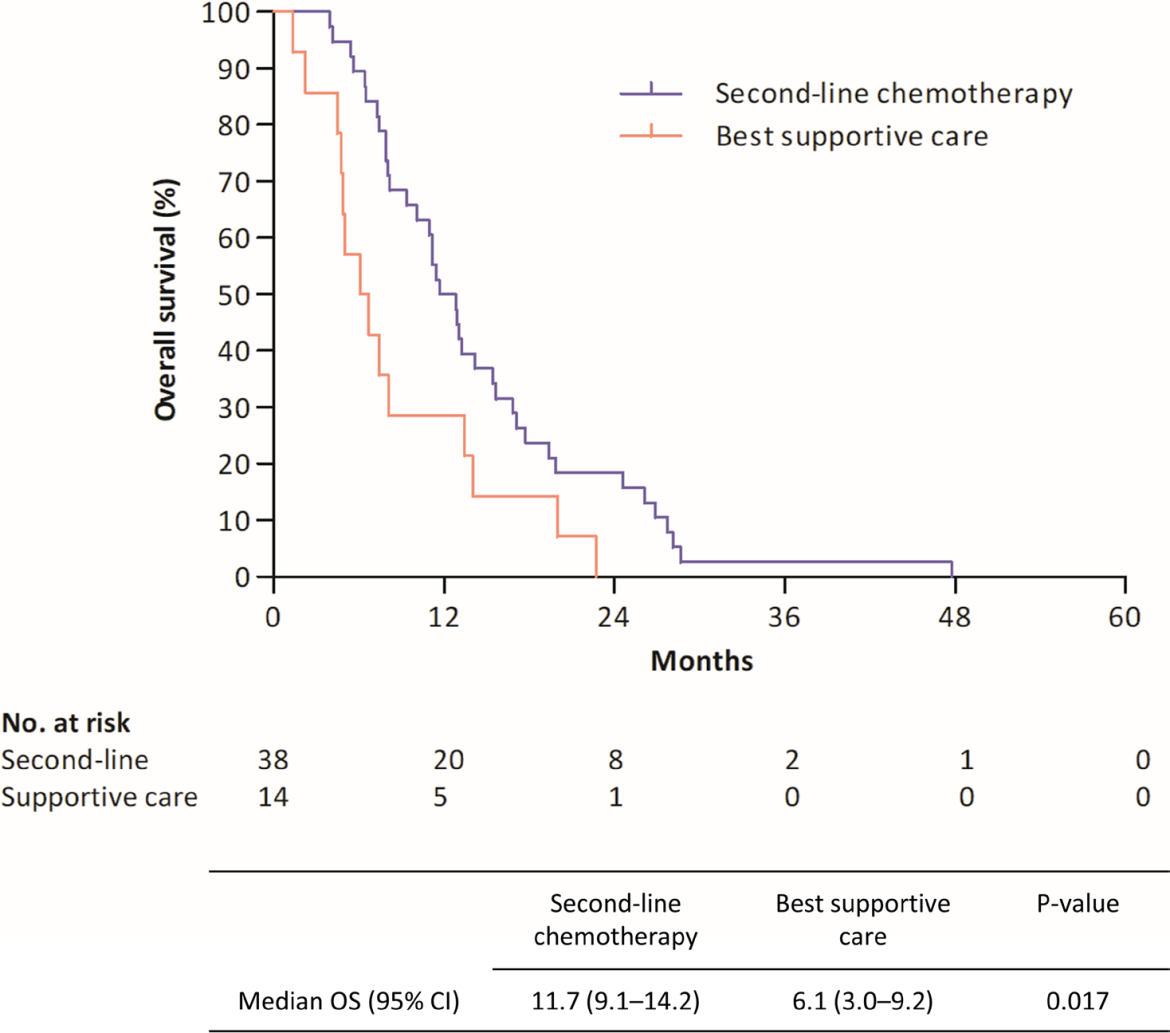
Survival analysis according to subsequent chemotherapy among patients for whom the survival data collection was completed. OS, overall survival; 95% CI, 95% confidence interval.

Among the patients who survived after progression on first‐line atezolizumab combined with chemotherapy (*n* = 78), 71.8% (*n* = 56) underwent second‐line treatment (Figure S3). Topoisomerase I inhibitors accounted for 71.4% of the second‐line treatments, followed by paclitaxel (23.2%). Among the patients who survived and experienced disease progression following the second‐line therapy (*n* = 38), 60.5% (*n* = 23) received third‐line chemotherapy. The median PFS2 of patients who received second‐line chemotherapy was 11.7 months (95% CI 9.2–14.1), whereas the median PFS of second‐line treatment was 4.8 months (95% CI 2.9–6.6). No significant difference in survival rates or PFS2 was observed according to the second‐line regimen (Figures S4 and S5). Among the patients whose survival data collection was completed, those who received subsequent therapy appeared to achieve a longer OS than those who only received best supportive care (median OS 11.7 vs. 6.1 months; Figure 4).

### 3.6 Safety

Safety assessment was conducted for all patients. After the interim analysis, an additional five adverse events were reported; however, none were related to the immunotherapy. The results of our safety analysis, as published previously, are shown in Table S2.

## 4 Discussion

The current study provides an updated analysis of the first multicenter prospective cohort study conducted in a real‐world clinical setting to evaluate the effectiveness and safety of first‐line atezolizumab combined with chemotherapy among patients with ES‐SCLC. Notably, our patients had a median PFS and OS of 6.2 and 17.1 months, respectively, with favorable outcomes observed in those who achieved SD or PR and longer PFI. Thoracic RT during first‐line atezolizumab had the potential to prolong OS in patients who responded to systemic treatment. Brain RT in patients with BM and the implementation of subsequent therapy after progression were identified as favorable prognostic factors for OS. These findings indicate that, even in an unselected population representative of actual clinical practice, survival outcomes were comparable to those observed in previous randomized trials. This demonstrates the real‐world applicability of this regimen and provides insights into individualized treatment strategies that may optimize patient management beyond the scope of prior trials.

Atezolizumab combined with chemotherapy has demonstrated promising effectiveness in real‐world settings, with a median OS of 11.3–15.5 months [5, 6, 7, 8, 9, 10, 13], surpassing the results of the IMpower133 study. Recent real‐world studies published after our earlier interim analyses have consistently shown favorable outcomes for first‐line immunotherapy regimens. In particular, a retrospective multicenter study conducted in France (*n* = 57) by Ezzedine et al. reported a median OS of 15.47 months. Similarly, a single‐center retrospective analysis in China (*n* = 75) demonstrated a median PFS and OS of 7.33 and 14.33 months, respectively [10]. These observed encouraging outcomes, even in cohorts including older patients or those with an ECOG PS of 2 or higher, could be explained by several hypotheses, such as the deliberate use of local treatments (e.g., brain and thoracic RT) and the administration of atezolizumab beyond progression [14]. Additionally, a real‐world retrospective study on immunotherapy in ES‐SCLC (*n* = 165) identified thoracic RT as a favorable prognostic factor for OS [9], whereas another retrospective study (*n* = 36) highlighted the beneficial therapeutic effects of combining atezolizumab with local ablative RT after progression [15]. Our study identified thoracic RT and brain RT for patients with BM as potential favorable prognostic factors, further supporting these hypotheses.

One notable finding of the current study was that although our previous study identified thoracic RT as a favorable prognostic factor for OS, the current analysis did not recognize thoracic RT as a significant factor. Instead, this study identified thoracic RT as a potentially favorable prognostic factor for long‐term responders to immunochemotherapy; meanwhile, thoracic RT provided survival benefits to patients who achieved any kind of tumor response to first‐line atezolizumab. Although the lack of statistical significance may be explained by the limited sample size—as supported by a post hoc power analysis showing a two‐sided power of approximately 40% at *α* = 0.05—this finding also suggests that thoracic RT may provide benefit only in selected patient populations. Numerous previous real‐world cohort studies have already identified thoracic RT combined with first‐line immunochemotherapy as a favorable prognostic factor for ES‐SCLC [5, 7, 9, 16]. The mechanisms underlying these findings include the enhancement of the anti‐tumor immune response through the combination of immunotherapy and RT [17], as well as the effects of RT in inducing antigen presentation, recruiting T cells to tumor sites, and altering the tumor microenvironment [18]. Furthermore, several studies recommend considering thoracic RT in ES‐SCLC after achieving systemic disease control [2, 19, 20], whereas others have demonstrated that thoracic RT promoted survival benefits regardless of tumor response to systemic therapy, particularly in patients without brain or liver involvement or with a lower tumor burden [19, 21, 22]. However, safety concerns remain. The phase II, multicenter, randomized TREASURE trial (NCT04462276), which evaluated first‐line atezolizumab–chemotherapy with thoracic RT in ES‐SCLC, halted enrollment after safety monitoring identified an imbalance in grade 5 severe adverse events in the RT arm; interim reports did not indicate a novel safety signal attributable to atezolizumab or thoracic RT, and detailed adverse‐event categories have yet to be publicly specified [23, 24]. Accordingly, we suggest that thoracic RT should be considered in selected patients based on tumor response to immunochemotherapy, tumor burden, and the risk of RT‐related adverse events. Nonetheless, further studies are warranted to enhance the definition of the appropriate indications for thoracic RT in ES‐SCLC.

The current study found that newly diagnosed or treatment‐emergent BM did not affect OS; however, these findings contradict the results of our previous interim analysis, which showed that BM was a poor prognostic factor for OS (adjusted HR, 2.143, 95% CI 1.058–4.339; *p* = 0.034) [11]. This discrepancy could be explained by the effect of brain RT in SCLC patients with BM. As shown in the current study, administering brain RT to patients with BM prolonged survival when compared to systemic therapy alone. BM has been widely recognized as a poor prognostic factor in SCLC. Similarly, the IMpower133 and CASPIAN studies also found that immunochemotherapy did not demonstrate a survival benefit in patients with BM [3, 4, 25]. However, growing evidence exists that supports the benefits of brain RT in SCLC patients with BM. In particular, Huang et al. reported that combining immunotherapy with WBRT improved the survival of SCLC patients with BM (*n* = 109) when compared to WBRT alone [26]. Similarly, Que et al. showed that among 187 patients with BM from SCLC, those receiving asynchronous brain RT and immunotherapy exhibited significantly longer OS than those receiving immunotherapy alone, with manageable treatment‐related adverse events [27]. Therefore, we believe that the application of brain RT in patients with BM might have mitigated the negative prognostic impact of BM on OS in this updated analysis.

The current study also analyzed the patterns and outcomes of subsequent treatment. Interestingly, those who received subsequent chemotherapy had a longer median OS than those who did not. These findings align with previous studies showing that subsequent therapy yielded better survival outcomes than best supportive care alone [28]. Thus, we analyzed the baseline characteristics between the groups receiving subsequent treatment and best supportive care and found no significant differences in patient characteristics, such as age, ECOG PS, and BM, between the two groups (Table S3). This finding indicates that although patients possessed similar baseline characteristics, patients in our cohort who received subsequent therapy appeared to achieve improved survival outcomes. However, this association should be interpreted with caution and not as evidence of a causal benefit. Since this study primarily focused on first‐line outcomes and clinical data were not systematically collected at the initiation of each subsequent therapy, there remains a potential for immortal time bias and performance status‐driven treatment selection bias. Therefore, the observed survival advantage associated with subsequent chemotherapy should be interpreted with caution as an observed association while acknowledging the associated potential clinical relevance in real‐world practice.

Regarding subsequent regimens, our study found no prognostic difference between topoisomerase I inhibitors and paclitaxel. However, rechallenge of platinum‐doublet based on prior PFI [2, 29] or lurbinectedin (although not widely used during the study period in South Korea due to lack of reimbursement) [30], could also be a viable option depending on the condition of the patient.

Compared to the pre‐immunochemotherapy era for first‐line treatment of SCLC, the current study found promising PFS2. Currently, several studies have investigated the PFS2 of ES‐SCLC patients treated with first‐line immunochemotherapy. Previous studies have reported that second‐line treatment following immunochemotherapy resulted in a PFS ranging from 3.4 to 5.7 months [29, 31], whereas second‐line treatment following platinum‐doublet therapy resulted in a PFS ranging from 3.0 to 3.4 months [32, 33]. Although the reasons for the favorable PFS2 after immunochemotherapy remain unclear, recent studies suggest that prior exposure to immunotherapy may increase the pool of T cells, which act as a chemosensitizer and provide a synergistic advantage for subsequent chemotherapy, potentially enhancing the efficacy of the treatment [34]. This evidence further supports the benefits of applying a successive treatment.

This study has several limitations. First, the cohort size was limited to 100 patients, which may have reduced the statistical power of the subgroup analyses. However, real‐world prospective studies on first‐line chemoimmunotherapy for ES‐SCLC remain scarce, and this cohort still presents meaningful clinical data. Moreover, survival differences according to known prognostic factors such as ECOG performance status, best response, and PFI support the reliability of the data collected. Second, we did not distinguish between consolidative and palliative thoracic RT during data collection, which may have interfered with interpreting the effect of consolidative thoracic RT on survival outcomes. However, since most patients who received thoracic RT achieved a complete or partial best response (90.5%), while the subgroup analysis of patients who responded to systemic therapy showed a borderline significant survival benefit (*p* = 0.050), the beneficial effect of thoracic RT administered during first‐line treatment can reasonably be regarded as that of consolidative RT in a real‐world clinical setting [2, 19, 20]. Third, patient characteristics after disease progression were not collected because this evaluation was not part of our primary outcome, which limits the possibility of subgroup analyses of subsequent therapy. Further studies should include such data for a thorough analysis. Fourth, due to the aggressive disease course of ES‐SCLC and the frequent reliance on small tissue sampling techniques (e.g., endobronchial ultrasound‐guided transbronchial needle aspiration, bronchoscopic biopsy, or percutaneous needle biopsy), as well as the lack of reimbursement for next‐generation sequencing testing during the study period in Korea, biomarker and genomic data were unavailable for analysis. Therefore, future studies incorporating biomarker and genomic data are warranted to enhance clinical interpretation and to improve predictions of the immunotherapy outcomes. Finally, the patient population consisted solely of East Asians, which may limit the extrapolation of our data to the general population. Thus, additional multinational studies are needed.

In conclusion, this prospective cohort study provides real‐world evidence on the favorable effectiveness and safety of front‐line atezolizumab combined with chemotherapy for ES‐SCLC patients over an extended follow‐up period. In addition, our findings showed that local treatment (e.g., thoracic RT for patients who achieved response to systemic treatment and brain RT in patients with BM) and subsequent treatment after disease progression were associated with improved OS, highlighting the potential value of implementing these treatments for eligible patients.

## Author Contributions

Conceived and designed the analysis: Yechan Song, Myeong Geun Choi, Yeon Joo Kim, Jae Cheol Lee, Wonjun Ji, In‐Jae Oh, Sung Yong Lee, Seong Hoon Yoon, Shin Yup Lee, Jeong Eun Lee, Eun Young Kim, Ho Young Kim, and Chang‐Min Choi. Collected the data: Yechan Song, Myeong Geun Choi, and Chang‐Min Choi. Contributed data or analysis tools: Yechan Song, Myeong Geun Choi, Yeon Joo Kim, Jae Cheol Lee, Wonjun Ji, In‐Jae Oh, Sung Yong Lee, Seong Hoon Yoon, Shin Yup Lee, Jeong Eun Lee, Eun Young Kim, Ho Young Kim, and Chang‐Min Choi. Performed the analysis: Yechan Song, Myeong Geun Choi, and Chang‐Min Choi. Wrote the paper: Yechan Song, Myeong Geun Choi, and Chang‐Min Choi. Supervision: Jae Cheol Lee and Chang‐Min Choi. Writing – review and editing: Yechan Song, Myeong Geun Choi, Yeon Joo Kim, Jae Cheol Lee, Wonjun Ji, In‐Jae Oh, Sung Yong Lee, Seong Hoon Yoon, Shin Yup Lee, Jeong Eun Lee, Eun Young Kim, Ho Young Kim, and Chang‐Min Choi.

## Funding

This work was supported by the Roche.

## Ethics Statement

This prospective cohort study was approved by the institutional review board (IRB) of Asan Medical Center (IRB No. 2021‐1237) and was registered by the clinical research information service (Registration No. KCT0006818). Informed consent was obtained from all participants before enrollment. Finally, the trial was designed and conducted in accordance with the Helsinki Declaration and the Ethical Guidelines for Clinical Studies.

## Conflicts of Interest

The authors declare no conflicts of interest.

## Supporting information


**Data S1:** tca70201‐sup‐0001‐Supinfo.docx.

## Data Availability

The data that support the findings of this study are available on request from the corresponding author. The data are not publicly available due to privacy or ethical restrictions.
