# Supplementary material for: Extended Follow‐Up Analysis of First‐Line Atezolizumab in Extensive‐Stage Small Cell Lung Cancer: A Real‐World Multicenter Prospective Cohort Study
Source: Thorac Cancer. 2025 Dec 11;16(23):e70201. doi: 10.1111/1759-7714.70201 (PMC12698942; doi:10.1111/1759-7714.70201)
Supplement: Supplementary file 1 — Data S1: tca70201‐sup‐0001‐Supinfo.docx. [file TCA-16-e70201-s001.docx]

**SUPPORTING INFORMATION**

**Title: Extended Follow-Up Analysis of First-Line Atezolizumab in Extensive-Stage Small Cell Lung Cancer: A Real-World Multicenter Prospective Cohort Study**

Yechan Song^1*^, Myeong Geun Choi^2*^, Yeon Joo Kim^3^, Jae Cheol Lee^4^, Wonjun Ji^1^, In-Jae Oh^5^, Sung Yong Lee^6^, Seong Hoon Yoon^7^, Shin Yup Lee^8^, Jeong Eun Lee^9^, Eun Young Kim^10^, Ho Young Kim^11^, Chang-Min Choi^1,4^**^†^**

^1^ Department of Pulmonary and Critical Care Medicine, Asan Medical Center, University of Ulsan College of Medicine, Seoul

^2^ Division of Pulmonary and Critical Care Medicine, Department of Internal Medicine, Ewha Womans University Mokdong Hospital, Ewha Womans University College of Medicine, Seoul

^3^ Department of Pulmonology, Nowon Eulji Medical Center, Eulji University School of Medicine, Seoul

^4^ Department of Oncology, Asan Medical Center, University of Ulsan College of Medicine, Seoul

^5^ Department of Internal Medicine, Chonnam National University Hwasun Hospital, Chonnam National University Medical School, Hwasun

^6^ Division of Pulmonary, Allergy, and Critical Care Medicine, Department of Internal Medicine, Korea University Guro Hospital, Korea University College of Medicine, Seoul

^7^ Division of Pulmonology, Allergy, and Critical Care Medicine, Department of Internal Medicine, Pusan National University Yangsan Hospital, Yangsan

^8^ Department of Internal Medicine, Kyungpook National University, School of Medicine, Daegu

^9^ Division of Pulmonology, Department of Internal Medicine, Chungnam National University College of Medicine, Daejeon

^10^ Division of Pulmonary and Critical Care Medicine, Department of Internal Medicine, Severance Hospital, Yonsei University College of Medicine, Seoul, Korea

^11^ Division of Hematology and Oncology, Department of Internal Medicine, Hallym University Sacred Heart Hospital, Anyang, Republic of Korea

^*^ Both authors contributed equally to this study.

**^†^Corresponding Author:**

**Chang-Min Choi, MD, PhD**

Department of Pulmonary and Critical Care Medicine, Asan Medical Center,

University of Ulsan College of Medicine, 88, Olympic-ro 43-gil, Songpa-gu,

Seoul 05505, South Korea

Tel.: 82-2-3010-5902

Fax: 82-2-3010-6968

E-mail: [ccm@amc.seoul.kr](mailto:ccm@amc.seoul.kr)

**Table S1.** Baseline characteristics.

| **Variables** | **Total patients**  **(n = 100)** | **IMpower133**  **(n = 201)** |
| --- | --- | --- |
| Age (year), median (range) | 69 (47–90) | 64 (28–90) |
| Age group (years) |  |  |
| <65 | 24 (24) | 111 (55.2) |
| 65–74 | 51 (51) | 71 (35.3) |
| ≥75 | 25 (25) | 19 (9.5) |
| Sex, male | 92 (92) | 129 (64.2) |
| ECOG PS |  |  |
| 0–1 | 94 (94) | 201 (100) |
| 2 | 5 (5) | 0 |
| 3 | 1 (1) | 0 |
| Smoking history |  |  |
| Never | 11 (11) | 9 (4.5) |
| Current smoker | 48 (48) | 74 (36.8) |
| Ex-smoker | 40 (40) | 118 (58.7) |
| Unknown | 1 (1) | 0 |
| Brain metastasis at diagnosis | 26 (26) | 17 (8.5) |

Abbreviations: ECOG PS, Eastern Cooperative Oncology Group Performance Status.

**Figure S1.** Overall survival according to (A) age, (B) Eastern Cooperative Oncology Group Performance Status, (C) best response, and (D) platinum-free interval.


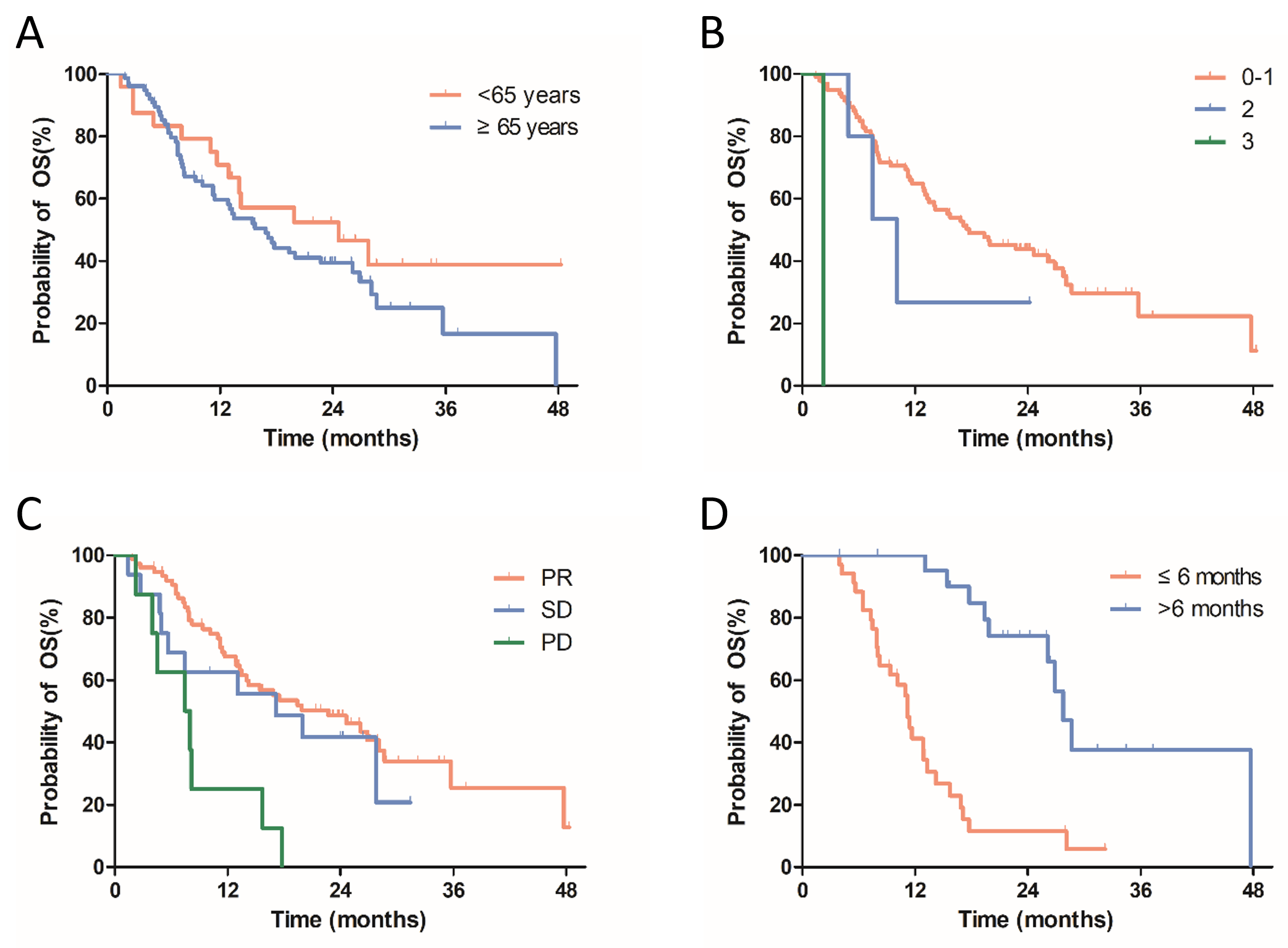


|  | Median OS, months (95% CI) | p-value |
| --- | --- | --- |
| Age (years) |  |  |
| <65 | 24.6 (8.9–40.3) | 0.227 |
| ≥65 | 16.8 (12.0–21.6) |  |
| ECOG PS |  |  |
| 0–1 | 17.7 (11.0–24.5) | <0.001 |
| 2 | 10.1 (5.8–14.4) |  |
| 3 | 2.2 (N/E–N/E) |  |
| Best response |  |  |
| PR | 19.8 (10.2–29.5) | 0.001 |
| SD | 17.1 (4.6–29.6) |  |
| PD | 7.4 (2.5–12.3) |  |
| PFI |  |  |
| ≤6 months | 11.2 (9.5–12.9) | <0.001 |
| >6 months | 27.7 (25.0–30.4) |  |

Abbreviations: CI, confidence interval; N/E, not evaluated; ECOG PS, Eastern Cooperative Oncology Group Performance Status; PR, partial response; SD, stable disease; PD, progressive disease; PFI, platinum-free interval.

**Figure S2.** Progression-free survival according to (A) age, (B) Eastern Cooperative Oncology Group Performance Status, (C) best response, and (D) thoracic radiotherapy during first-line chemotherapy.


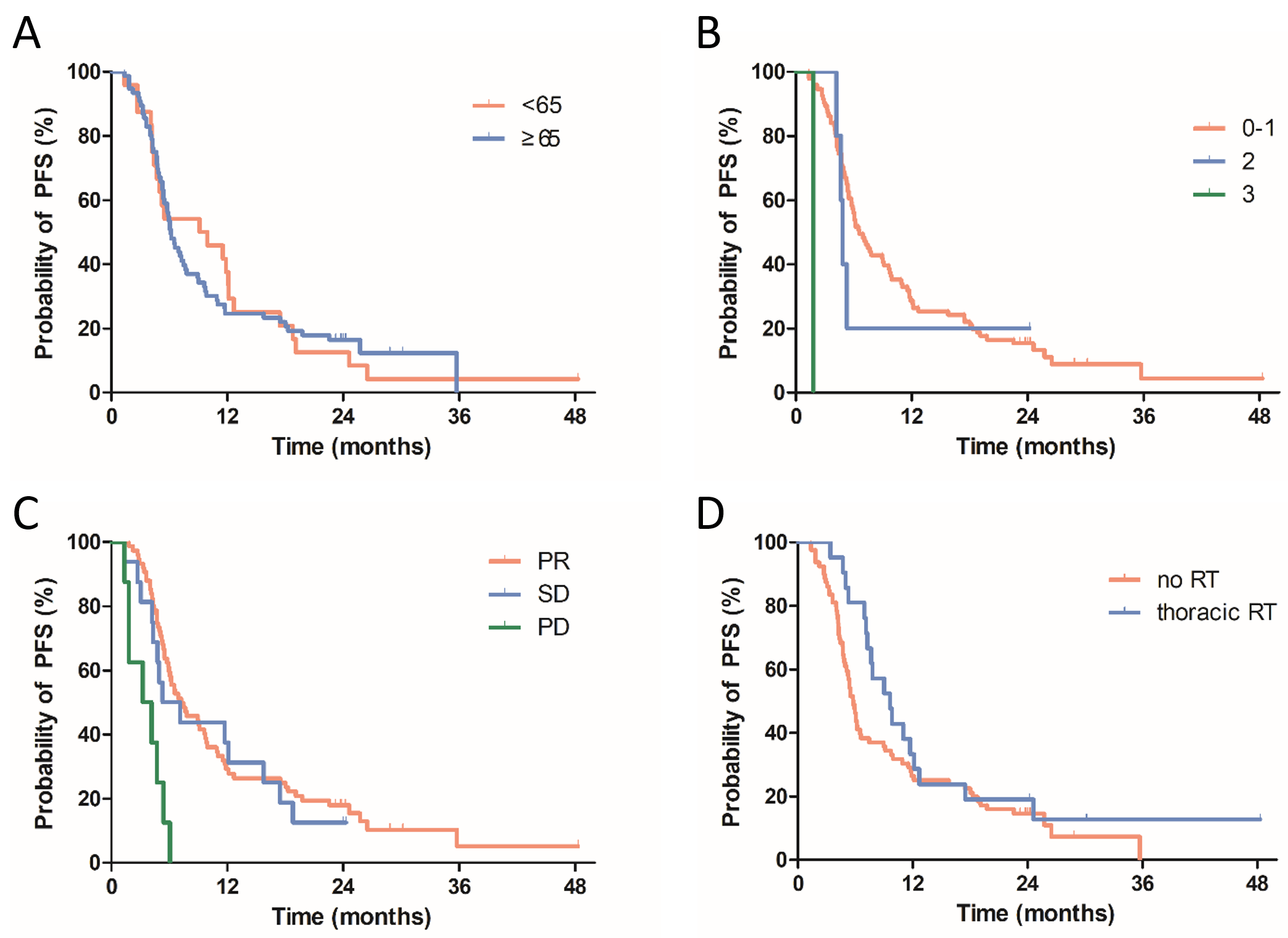


|  | Median PFS, months (95% CI) | p-value |
| --- | --- | --- |
| Age (years) |  |  |
| <65 years | 9.1 (1.5–16.7) | 0.926 |
| ≥65 years | 6.1 (5.1–7.2) |  |
| ECOG PS |  |  |
| 0–1 | 6.6 (5.1–8.0) | <0.001 |
| 2 | 4.8 (4.5–5.2) |  |
| 3 | 1.8 (N/E– N/E) |  |
| Best response |  |  |
| PR | 7.2 (5.6–8.9) | <0.001 |
| SD | 5.3 (1.1–9.5) |  |
| PD | 3.2 (0.5–6.4) |  |
| Thoracic RT during first-line chemotherapy |  | 0.150 |
| No RT | 5.8 (5.1–6.5) |  |
| Thoracic RT | 9.6 (6.5–12.7) |  |

Abbreviations: CI, confidence interval; ECOG PS, Eastern Cooperative Oncology Group Performance Status; N/E, not evaluated; RT, radiotherapy; PR, partial response; SD, stable disease; PD, progressive disease.

**Table S2.** Adverse events related to treatment (n = 100).

| **AEs** | **AEs of all grades** | **Grade ≥3** | **Grade 5** |
| --- | --- | --- | --- |
| **Treatment-related AEs** | 56 (56.0) | 7 | 2 |
| **Treatment-related AEs occurring in ≥2% or with grade ≥3** | | | |
| Anorexia | 9 (9.0) | 0 | 0 |
| Neutropenia | 8 (8.0) | 1 (1.0) | 1 (1.0) |
| General weakness | 6 (6.0) | 0 | 0 |
| Skin rash | 5 (5.0) | 0 | 0 |
| Pruritus | 5 (5.0) | 0 | 0 |
| Nausea | 5 (5.0) | 0 | 0 |
| Elevated ALT | 4 (4.0) | 0 | 0 |
| Vomiting | 4 (4.0) | 0 | 0 |
| Constipation | 4 (4.0) | 0 | 0 |
| Pneumonia | 3 (3.0) | 1 (1.0) | 1 (1.0) |
| Elevated AST | 3 (3.0) | 1 (1.0) | 0 |
| Cough | 3 (3.0) | 0 | 0 |
| Hyponatremia | 3 (3.0) | 0 | 0 |
| Peripheral neuropathy | 2 (2.0) | 0 | 0 |
| Hypothyroidism | 2 (2.0) | 0 | 0 |
| Fatigue | 2 (2.0) | 0 | 0 |
| Hyperglycemia | 2 (2.0) | 1 (1.0) | 0 |
| Hematemesis | 1 (1.0) | 1 (1.0) | 0 |
| Paralytic ileus | 1 (1.0) | 1 (1.0) | 0 |
| Pleural effusion | 1 (1.0) | 1 (1.0) | 0 |
| Enterocolitis | 1 (1.0) | 0 | 0 |
| **Immune-related AEs** |  | 0 | 0 |
| Skin rash | 6 (6.0) | 0 | 0 |
| Hepatitis | 4 (4.0) | 1 (1.0) | 0 |
| Peripheral neuropathy | 3 (3.0) | 0 | 0 |
| Diabetes | 3 (3.0) | 0 | 0 |
| Pneumonitis | 2 (2.0) | 0 | 0 |
| Hypothyroidism | 2 (2.0) | 0 | 0 |
| Colitis | 1 (1.0) | 1 (1.0) | 0 |
| Adrenal insufficiency | 1 (1.0) | 0 | 0 |

Abbreviations: AE, adverse event; ALT, alanine transaminase; AST, aspartate transaminase.

**Figure S3.** Subsequent treatment pattern.

Abbreviations: CAV, cyclophosphamide, doxorubicin, vincristine.

**Figure S4.** Survival analysis according to the second-line chemotherapy regimen.


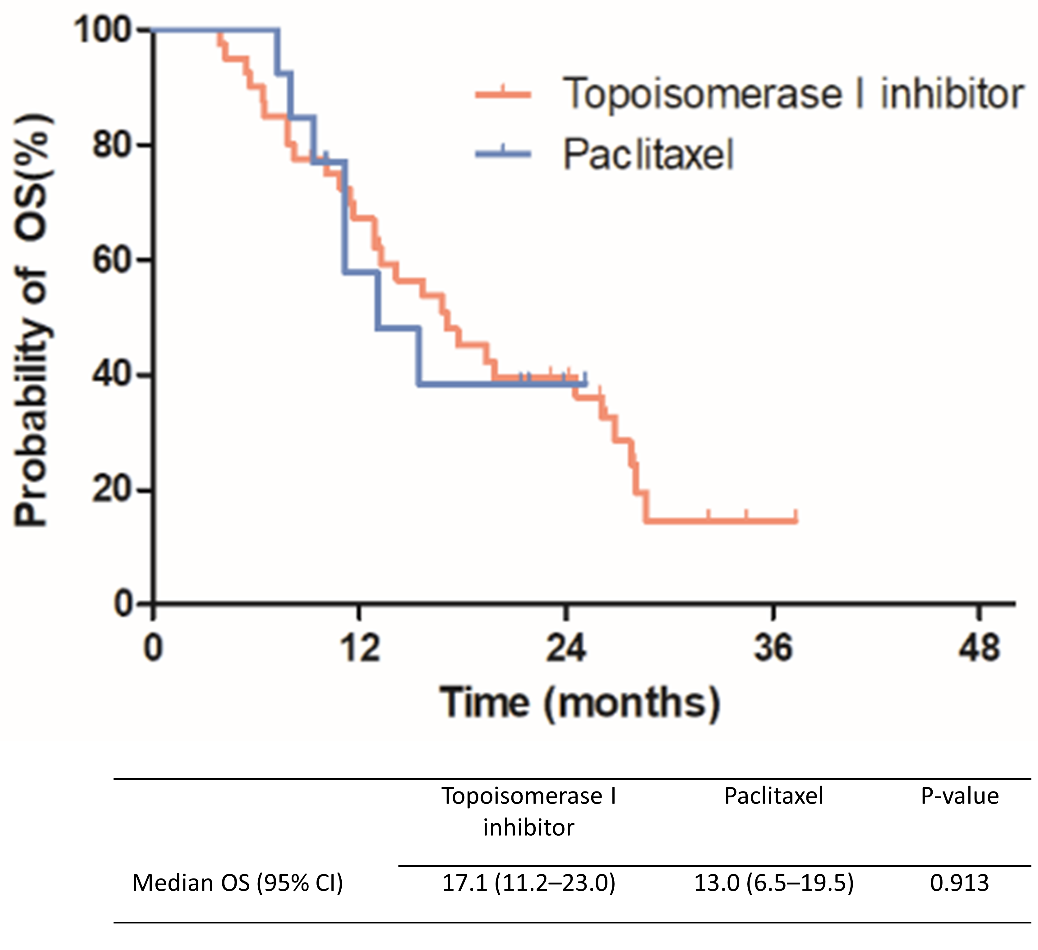


**Figure S5.** Kaplan–Meier curves for (A) time to second objective disease progression-free survival (PFS2) and (B) PFS2 according to second-line chemotherapy regimen.

**
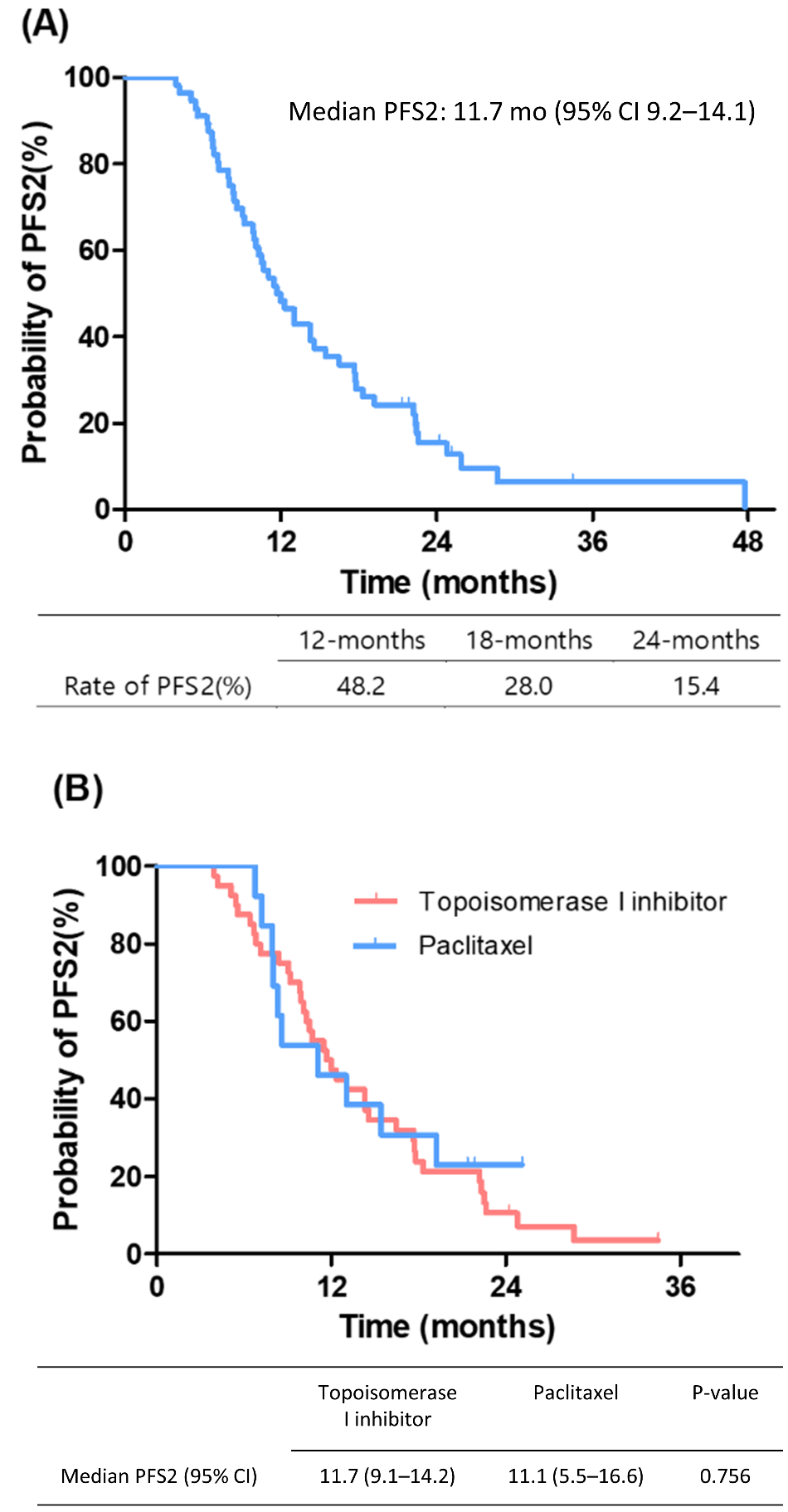
**

**Table S3**. Baseline characteristics according to subsequent treatment among patients for whom survival data collection was completed.

|  | Subsequent chemotherapy  (n = 38) | Best supportive care  (n = 14) | p-value |
| --- | --- | --- | --- |
| Age (mean, years) | 69.6 | 71.7 | 0.394 |
| Smoking (n, %) |  |  |  |
| Never smoker | 5 (13.2) | 2 (14.3) | 1.000 |
| Ever smoker | 33 (86.8) | 12 (85.7) |  |
| ECOG PS (n, %) |  |  |  |
| 0–1 | 37(97.4) | 11 (78.6) | 0.055 |
| 2≤ | 1 (2.6) | 3 (21.4) |  |
| Brain metastasis at diagnosis (n, %) |  |  |  |
| Absent | 32 (84.2) | 8 (57.1) | 0.063 |
| Present | 6 (15.8) | 6 (42.9) |  |

Abbreviations: ECOG PS, Eastern Cooperative Oncology Group Performance Status.
